# Misracialization of Indigenous people in population health and mortality studies: a scoping review to establish promising practices

**DOI:** 10.1093/epirev/mxad001

**Published:** 2023-04-06

**Authors:** Danielle R Gartner, Ceco Maples, Madeline Nash, Heather Howard-Bobiwash

**Affiliations:** Department of Epidemiology and Biostatistics, College of Human Medicine, Michigan State University, East Lansing, MI 48824, United States; Department of Anthropology, College of Social Science, Michigan State University, East Lansing, MI 48824, United States; Department of Sociology, College of Social Science, Michigan State University, East Lansing, MI 48824, United States; Department of Anthropology, College of Social Science, Michigan State University, East Lansing, MI 48824, United States

**Keywords:** Indigenous, misclassification, American Indian/Alaska Native, Native American, mortality, morbidity, health, classification

## Abstract

Indigenous people are often misracialized as other racial or ethnic identities in population health research. This misclassification leads to underestimation of Indigenous-specific mortality and health metrics, and subsequently, inadequate resource allocation. In recognition of this problem, investigators around the world have devised analytic methods to address racial misclassification of Indigenous people. We carried out a scoping review based on searches in PubMed, Web of Science, and the Native Health Database for empirical studies published after 2000 that include Indigenous-specific estimates of health or mortality and that take analytic steps to rectify racial misclassification of Indigenous people. We then considered the weaknesses and strengths of implemented analytic approaches, with a focus on methods used in the US context. To do this, we extracted information from 97 articles and compared the analytic approaches used. The most common approach to address Indigenous misclassification is to use data linkage; other methods include geographic restriction to areas where misclassification is less common, exclusion of some subgroups, imputation, aggregation, and electronic health record abstraction. We identified 4 primary limitations of these approaches: (1) combining data sources that use inconsistent processes and/or sources of race and ethnicity information; (2) conflating race, ethnicity, and nationality; (3) applying insufficient algorithms to bridge, impute, or link race and ethnicity information; and (4) assuming the hyperlocality of Indigenous people. Although there is no perfect solution to the issue of Indigenous misclassification in population-based studies, a review of this literature provided information on promising practices to consider.

## Introduction

Comprehensive and interpretable estimates of morbidity and mortality to guide public policy and resource allocation require an accurate ascertainment of population. Unfortunately, in Canada, Australia, New Zealand, and the United States, the seemingly simple task of counting and grouping Indigenous people to portray an accurate picture of mortality and morbidity is not straightforward. Racial misclassification, or misracialization, of Indigenous people in vital records,^1^ administrative health records,^2^^–^^7^ surveillance,^8^^,^^9^ and censuses^10^ is a well-documented phenomenon, with estimates suggesting 18% of American Indian/Alaska Natives (AI/AN), for example, are misclassified in death certificate data as another race.^11^ In Michigan, cancer case counts misclassify more than 50% of AI/AN people.^12^ Misclassification leads to undercounting of Indigenous people, underestimation of disease or mortality burden, and therefore, inadequate resource allocation. This issue has led to substantial efforts to analytically correct these inaccuracies in secondary data analyses.^13^

Several commentaries have identified the issue of Indigenous erasure through racial misclassification in health data, describing how these challenges exist because they are rooted in the ways the power to define who is Indigenous is aligned with settler-colonial-state power. Some offer critiques of the analytic methods applied to address Indigenous erasure, and note the complexity and nuance of this issue.^8^^,^^14^^–^^21^ Yet, to our knowledge, in no published reports have the suite of methods used to address misclassification through a systematic approach been analyzed (see Thompson et al.^21^ for an Australia-based study of data linkages). The purpose of this scoping review is to consider the strengths and weaknesses of the analytic approaches used to address Indigenous misclassification in population-level mortality and health studies. Using this information, we identify considerations that might guide future efforts to quantify Indigenous mortality and health. Although we identify and discuss articles about areas outside of the United States, our focus is primarily on studies within the US borders. Furthermore, critique of the settler-colonial logics used to identify Indigenous people has long been undertaken in fields outside of epidemiology. We incorporate this literature to inform our interpretations while recognizing we cannot do this large literature and history of thought the justice it deserves in this single article.

## Background

Classifying Indigenous populations as a racial group poses numerous challenges. Race is a sociopolitical construction and, therefore, varies considerably around the world. Classification and categorization necessitate defining groups, a seemingly impossible task considering there are approximately 476.6 million Indigenous people living around the world, comprising 6% of the population.^23^ As a group, Indigenous people are not monolithic. In recognition of this vast diversity, the United Nations recommends using criteria that help Indigenous people self-identify, rather than defining them, and offer this articulation:

Indigenous communities, peoples and nations are those which, having a historical continuity with pre-invasion and pre-colonial societies that developed on their territories, consider themselves distinct from other sectors of the societies now prevailing on those territories, or parts of them. They form at present non-dominant sectors of society and are determined to preserve, develop and transmit to future generations their ancestral territories, and their ethnic identity, as the basis of their continued existence as peoples, in accordance with their own cultural patterns, social institutions and legal system.^24^^(p29)^

The Indigenous populations included in this scoping review have undergone racialization by the settler states that occupy their lands or territories, and although the United States, Canada, Australia, and New Zealand may legislate Indigenous identity differently, they have historically used similar strategies to “eliminate the Native” and dispossess Indigenous people of their lands.^25^ These populations share similar experiences of oppression, domination, and discrimination by and within nation-states. In the United States, the commonly used racial category for Indigenous Americans is “American Indian or Alaska Native,” which has been defined by the Office of Management and Budget (OMB) as “[a] person having origins in any of the original peoples of North and South America (including Central America), and who maintains tribal affiliation or community attachment.”^26^ This definition is operationalized in several ways and sometimes differently in numerator and denominator data, which has implications for epidemiologic studies. One of the tactics that settler-colonial states have used, and continue to use to eliminate Indigenous people, is population transfer through accounting.^27^ Settler-colonial governments often fail to count, or consistently undercount, Indigenous populations, thereby rendering them invisible. Misclassification and, in some cases, the methods used to address misclassification contribute to this elimination.

## Methods

Using the Preferred Reporting Items for Systematic Reviews and Meta-Analyses (PRISMA) extension for scoping reviews checklist,^28^ we read and synthesized the peer-reviewed epidemiologic literature regarding racial misclassification of Indigenous people. Articles were pulled from 3 databases: PubMed, Native Health Database, and Web of Science. Articles had to be in English; report on an empirical study; focus on health, disease, or death outcomes; focus on Indigenous people; and analytically address misclassification. Articles on research that focused on misclassification but did not use methods to fix misclassification were excluded. We excluded articles published before 2000 to better capture the analytic techniques more commonly used today, such as those facilitated by increased computing power.

Our first searches took place on February 1, 2022, in PubMed. Searches included specific terms relating to our research questions. These terms, nonextensively, were as follows: “American Indian,” “Alaskan Native,” “Indigenous,” and “misclassification.” We were able to use an additional filter in the Web of Science database search to exclude nonempirical articles (i.e., letters and editorial materials). A general search in Web of Science, for example, included: (“native american” or “alaskan” or “american indian” or “indigenous” or “aboriginal” or “Pacific islander” or “Native Hawaiian”) AND (“misclassification” or “misestimation” or “underestimation” or misclass^*^) AND (“health” or “disease” or “wellbeing” or “mortality” or “morbidity”). We compiled identifying article information (e.g., title and PubMed identifier) from each search. Duplicates were identified using a spreadsheet filter.

After removing duplicates, 2 co-authors (D.R.G., C.M.) screened articles by reviewing the titles and abstracts. Articles that did not meet inclusion criteria were excluded. When there was disagreement, articles were flagged for further review by other coauthors (H.H.-B., M.N.) for a final determination. To determine if the articles were eligible to be included in this review, we read the full text of included articles. We then reviewed those article’s references and added additional articles that fit our inclusion criteria.

We extracted relevant data from each article into a spreadsheet. All coauthors read an initial set of representative articles to determine relevant data to extract. We noted the database and reviewing coauthor. Additional information extracted included title, journal, first author, publication year, data source(s), outcome(s) studied, Indigenous classification definition, geographic location, comparison group, methods used to address misclassification, and results. We also noted problems with the misclassification approach. Columns were added for additional information that was too ambiguous to categorize. When unclear, group discussion determined what information would be entered into a column. Two coauthors (D.G., H.H.-B.) created initial categories to group (e.g., create categories for health outcomes) and quantify extracted information, with finalized categorizations determined with the full coauthor team. We noted frequencies and calculated the percentage of articles by category to summarize the primary extracted information.

## Results

**Figure 1:**
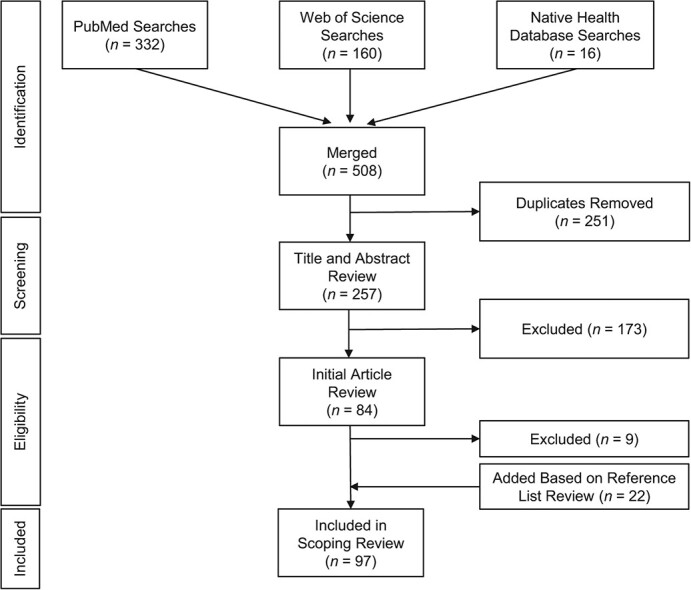
Preferred Reporting Items for Systematic Reviews and Meta-Analyses (PRISMA) flow chart of databases searched and studies included or excluded, including databases searched, exclusion criteria applied, and article counts. The flowchart is based on the PRISMA extension for scoping reviews.

After searching databases for relevant articles and removing duplicates, 257 articles were screened by title and abstract. Of these, 173 did not meet inclusion criteria. The remaining 84 full-text articles were assessed for eligibility and 9 were then excluded. Snowball methods, based on coauthors carrying out a scan of article references, led to the inclusion of 22 additional full-text articles. Overall, 97 articles were included in analyses (Figure 1). Table 1 lists extracted information from each article.

**Table 1:** Studies included in this scoping review and primary extracted data.

**First author, year^**reference**^**	**Country**	**Geographic location** ^**a**^	**Outcome of studied health issue**	**Misclassification approach**					
				**Linkage**	**IHS as linkage data set**	**Geographic restriction**	**Exclude Hispanic AI/AN**	**Create multiple estimates**	**Other**
Arias, 2021^84^	US	US	Mortality and life expectancy	X			X		
Arias, 2008^85^	US	US	Mortality and life expectancy	X					
Arias, 2016^86^	US	US	Mortality and life expectancy	X		X			
Arias, 2014^87^	US	US	Mortality and life expectancy	X	X	X	X		
Becker,2002^88^	US	Pacific Northwest Region	Morbidity: cancer	X	X				
Becker, 2008^89^	US	US	Morbidity: cancer	X	X	X			
Bertolli, 2007^90^	US	Multiple States; Los Angeles County, California	Morbidity: infectious disease	X	X				
Bigback, 2015^91^	US	Oregon and Washington	Morbidity: hospitalizations	X	X				
Blakely, 2002^92^	New Zealand	New Zealand	Mortality and life expectancy	X	n/a				
Bliss, 2008^93^	US	US	Morbidity: cancer	X	X	X			
Brandenburg, 2020^94^	US	Oklahoma	Mortality and life expectancy	X					Aggregation of data on AI/AN and White people
Briffa, 2010^95^	Australia	Western Australia	Morbidity: chronic disease/mortality and life expectancy	X	n/a				
Bruegl, 2020^96^	US	Pacific Northwest Region	Morbidity: cancer/mortality and life expectancy	X	X				
Cheek, 2014^97^	US	US	Mortality and life expectancy	X	X	X	X		
Cho, 2014^98^	US	US	Mortality and life expectancy	X	X	X	X		
Creswell, 2013^58^	US	Wisconsin	Morbidity: cancer	X					
Curtis, 2005^99^	New Zealand	New Zealand	Morbidity: cancer/mortality and life expectancy	X	n/a			X	
Dankovchik, 2015^100^	US	Pacific Northwest Region	Mortality and life expectancy	X	X				
Dankovchik, 2014^101^	US	Washington	Morbidity: injury	X	X				
Dougherty, 2019^102^	US	US	Mortality and life expectancy	X	X				
Draper, 2009^103^	Australia	Western Australia	Mortality and life expectancy	X	n/a				
Duke, 2019^104^	US	Eastern (& Southern) Region	Mortality and life expectancy	X	X				
Espey, 2014^13^	US	US	Mortality and life expectancy	X	X	X	X		
Espey, 2008^41^	US	US	Morbidity: cancer	X	X	X			
Espey, 2014^11^	US	US	Mortality and life expectancy	X	X	X	X		
Espey, 2005^105^	US	US	Mortality and life expectancy	X	X	X			
Espey, 2007^49^	US	US	Morbidity: cancer/mortality and life expectancy	X		X			
Espey, 2003^106^	US	US	Mortality and life expectancy			X			
Foo, 2021^60^	US	Alameda County, California	Morbidity: infectious disease/Mortality and life expectancy						EHR note review to identify Maya language speakers
Foote, 2007^107^	US	Wisconsin	Morbidity: cancer	X	X				
Graber, 2005^108^	US	Maine	Mortality and life expectancy	X	X				
Groom, 2014^109^	US	US	Mortality and life expectancy	X	X	X	X		
Haring, 2018^110^	US	US; Eastern Region; 9 counties in New York (Haudenosaunee Nation)	Mortality and life expectancy	X	X	X	X		
Harwell, 2002^111^	US	Montana	Mortality and life expectancy	X	X				
Hatcher, 2020^112^	US	Pacific Northwest Region	Mortality and life expectancy	X	X				
Haverkamp, 2008^113^	US	US	Mortality and life expectancy			X			
Henderson, 2008^114^	US	US	Morbidity: cancer	X	X				
Herne, 2016^115^	US	US	Mortality and life expectancy	X	X	X	X		
Herne, 2014^116^	US	US	Mortality and life expectancy	X	X	X	X		
Hoopes, 2010^117^	US	14 Counties in Washington	Morbidity: cancer	X	X				
Hoopes, 2015^118^	US	Washington	Mortality and life expectancy	X	X				
Hoopes, 2012^119^	US	Pacific Northwest Region	Morbidity: cancer/mortality and life expectancy	X	X				
Jackson, 2003^120^	US	Oregon	Morbidity: infectious disease	X	X				
Jacobs-Wingo, 2016^121^	US	US	Mortality and life expectancy	X	X	X	X		
Jarrín, 2020^55^	US	US	Morbidity: chronic disease	X					Imputation
Jim, 2008^122^	US	US	Morbidity: cancer	X	X	X			
Jim, 2014^1^	US	United States	Morbidity: cancer/mortality and life expectancy	X	X	X	X		
Johnson, 2009^123^	US	Michigan	Morbidity: cancer	X	X				
Joshi, 2018^124^	US	Washington	Mortality and life expectancy	X	X				
Joshi, 2019^125^	US	Washington	Mortality and life expectancy	X	X			X	
Korenbrot, 2003^35^	US	California	Morbidity: hospitalizations	X	X				
Labgold, 2021^56^	US	Fulton County, Georgia	Morbidity: infectious disease						Imputation
Landen, 2014^126^	US	US	Mortality and life expectancy	X	X	X	X		
Lee, 2009^127^	US	California	Morbidity: various					X	
Lemrow, 2008^128^	US	US	Morbidity: cancer	X	X	X			
Li, 2014^129^	US	US	Morbidity: cancer/mortality and life expectancy	X	X	X	X		
Madden, 2012^130^	Australia	Australia	Mortality and life expectancy	X	n/a				
Mak, 2008^131^	Australia	Western Australia	Morbidity: infectious disease	X	n/a				
Melkonian, 2020^132^	US	US	Morbidity: cancer	X	X	X	X		
Melkonian, 2020^133^	US	US	Morbidity: cancer	X	X	X			
Melkonian, 2019^134^	US	US	Morbidity: cancer	X	X	X	X		
Mowls, 2015^135^	US	Oklahoma	Morbidity: cancer	X	X				
Murphy, 2014^136^	US	United States	Mortality and life expectancy	X	X	X	X		
Paisano, 2003^137^	US	US	Mortality and life expectancy	X	X	X			
Perdue, 2008^138^	US	US	Morbidity: cancer	X	X	X			
Plescia, 2014^139^	US	US	Mortality and life expectancy	X	X	X	X		
Puukka, 2004^140^	US	Pacific Northwest Region	Morbidity: cancer	X	X				
Puukka. 2005^141^	US	Pacific Northwest Region	Morbidity: cancer	X	X			X	
Reichman, 2008^142^	US	US	Morbidity: cancer	X	X	X			
Reilley, 2014^143^	US	US	Mortality and life expectancy	X	X	X	X		
Rhoades, 2005^144^	US	US	Mortality and life expectancy						Use reported IHS counts and apply correction factor to correct for misclassification
Roen, 2014^12^	US	Michigan	Morbidity: cancer	X	X				
Sabatino, 2009^145^	US	US	Morbidity: cancer	X	X				
Sandiford, 2013^146^	New Zealand	Waitemata, New Zealand	Morbidity: prevention	X	n/a				
Schieb, 2014^147^	US	US	Mortality and life expectancy	X	X	X	X		
Singh, 2014^148^	US	US	Morbidity: cancer/mortality and life expectancy	X	X	X	X		
Stehr-Green, 2002^149^	US	Washington	Mortality and life expectancy	X	X				
Suryaprasad, 2014^150^	US	US	Mortality and life expectancy	X	X	X	X		
Swan, 2006^151^	US	California	Morbidity: prevention					X	Use several definitions of AI/AN and data sources
Thoroughman, 2002^152^	US	Oklahoma	Morbidity: infectious disease	X	X				
US Cancer Statistics Working Group, 2007^153^	US	US	Morbidity: cancer/mortality and life expectancy	X	X	X			
US Cancer Statistics Working Group, 2006^154^	US	US	Morbidity: cancer/mortality and life expectancy	X	X				
Watson, 2014^155^	US	US	Morbidity: cancer/mortality and life expectancy	X	X	X	X		
Weber, 2019^156^	US	Michigan	Morbidity: cancer	X	X				
Weinert, 2016^37^	US	Multiple States	Morbidity: hospitalizations						Use hospital data as denominator
Weir, 2008^157^	US	US	Morbidity: cancer	X	X	X			
White, 2014^158^	US	US	Morbidity: cancer/mortality and life expectancy	X	X	X	X		
White, 2014^159^	US	US	Mortality and life expectancy	X	X	X	X		
Wiggins, 2008^160^	US	US	Morbidity: cancer	X	X	X			
Wiggins, 2008^161^	US	US	Morbidity: cancer	X	X	X			
Wilson, 2008^162^	US	US	Morbidity: cancer	X	X	X			
Wingo, 2008^163^	US	US	Morbidity: cancer	X	X	X			
Wong, 2014^164^	US	US	Mortality and life expectancy	X	X	X	X		
Wood, 2014^165^	US	US	Morbidity: drug dependence					X	
Xu, 2012^166^	Australia	New South Wales, Australia	Morbidity: various	X	n/a				
Yankaskas, 2009^59^	US	North Carolina	Morbidity: cancer	X					
Young, 2015^167^	Canada	Ontario, Canada	Mortality and life expectancy		n/a				Ecological analysis based on Aboriginal population of residential area

Abbreviations: AI/AN, American Indian/Alaska Native; EHR, electronic health records; IHS, Indian Health Service; n/a, not applicable; US, United States.

^a^ Entire country, region, state, or tribe.

Of the 97 included articles, 9 (9%) were based outside of the current borders of the United States, representing Canada (*n* = 1, 11%), Australia (*n* = 5, 56%), and New Zealand (*n* = 3, 33%). Inclusion criteria limited studies to those that addressed misclassification in mortality (*n* = 41, 42%) or morbidity studies (*n* = 43, 44%), of which an additional 13% included estimates of both (*n* = 13 of 97). Estimated health outcomes are included in Table 2.

**Table 2:** Estimated mortality and morbidity outcomes, by study location.

**Study outcome**	**All studies** **(*n* = 97)**		**Non–US-based studies** **(*n* = 9)**	
	**No.**	**%**	**No.**	**%**
Mortality and life expectancy (*n* = 41)	41	42	4	44
Morbidity (*n* = 43)				
Cancer	28	29	0	0
Chronic disease	1	1	0	0
Infectious disease	5	5	1	11
Hospitalizations	3	3	0	0
Injury	1	1	0	0
Drug dependence	1	1	0	0
Prevention	2	2	1	11
Various^a^	2	2	1	11
Morbidity and cause-specific mortality (*n* = 13)				
Chronic disease	1	1	1	11
Infectious disease	1	1	0	0
Cancer	11	11	1	11

Abbreviation: US, United States

^a^ Includes studies with multiple health or disease outcomes. This category also includes 1 study in which the outcome was live birth.

In all but 12 studies, investigators used comparison groups defined by racial or ethnic identities (Table 3). Several studies named more than 1 comparison group, with most (*n* = 68, 70%) comparing results for Indigenous people with those for White people. Twelve percent of the studies specifically included comparisons between Indigenous and Black people. Nine studies (9%) described the comparison group in a generalized way as non-Indigenous. In contrast, 14 (14%) were specific in naming comparison groups, including comparisons between members of the same ethnicity in different regions. Ten studies made comparisons between groups without clearly qualifying the comparison in racial or ethnic terms, using, for example, the general term “all others.”

**Table 3:** Comparison groups by racial/ethnic identity.

**Race/ethnicity**	**All studies (*n* = 97)**	
	**No.**	**%** ^**a**^
White^b^	68	70
Black^c^	12	12
Other non-Indigenous^d^	9	9
Other specific^e^	14	14
Other nonspecific^f^	10	10
None or comparison not applicable	12	12

^a^ The percentages total more than 100% because studies may have multiple comparison groups.

^b^ Includes the following: European, American White, non-Hispanic White, Caucasian, non-Hispanic European American.

^c^ Includes the following: non-Hispanic Black, African American.

^d^ Includes the following variations: non-American Indian/Alaska Native, non-Indigenous, non-Māori, non-Pacific Islander, all other Americans combined; and uses main US Census categories to stand in for all others (White, Black, Asian/Pacific Islander, Hispanic, and others unknown).

^e^ Race or ethnicity of a specific group is stated (e.g., non-Mayan Latinx, US-born Asian/Pacific Islander, or foreign-born Asian/Pacific Islander) and may also include compared Indigenous peoples in contrasting regions or geographies (e.g., Wisconsin compared with all other Indigenous people in the United States; Haudenosaunee in New York State with Haudenosaunee in the United States overall, rural vs urban populations).

^f^ Race or ethnicity of the comparison group is not used, not clearly stated, or uses the term “all others” within the data set or registry without naming race or ethnicity.

Within the United States, 60% (*n* = 53 of 88) of studies created an estimate of mortality or health that was applicable to the entire nation, whereas 33% (*n* = 3 of 9) of non–US-based studies produced nationwide estimates (Table 4). It is important to note that not all counties or states are represented in the US-based data, because many US-wide studies include only a portion of states and counties within those states. A combined 16% (*n* = 14 of 88) of US-based studies included estimates specific to the Pacific Northwest region or a state within that region, representing the area of the United States where the most comprehensive health and mortality estimates have been created. Of note, there was just 1 US-based study in which a tribe-specific estimate was created.

**Table 4:** Geographic area of indigenous mortality or health estimate.

**Geographic area**	**Total no.**	**No.**	**%** ^**a**^
US-based studies	88		
Nationwide	53	53	60
Localized: region specific	11		
Pacific Northwest		8	9
Eastern (and Southern)		2	2
Multiple, not entire United States^b^		1	1
Localized: state specific	22		
California		3	3
Washington		5	6
Wisconsin		2	2
Michigan		3	3
Oklahoma		4	5
Maine		1	1
Oregon		1	1
North Carolina		1	1
Montana		1	1
Multiple^b^		1	1
Localized: county specific	5		
Washington		1	1
California		2	2
New York		1	1
Georgia		1	1
Localized: tribe specific	1		
Haudenosaunee		1	1
Non–US-based studies	9		
Nationwide	3	3	33
Localized: region specific	4		
Western Australia		3	33
New South Wales Australia		1	11
Localized: State or district specific	2		
Ontario, Canada		1	11
Waitemata, New Zealand		1	11
Localized: county specific			
Localized: Tribe specific			

Abbreviations: n/a, not applicable; US, United States

^a^ Percentage totals more than 100% because 2 studies created estimates specific to more than 1 geographic area.

^b^ Multiple includes studies in which multiple state-specific or county-specific estimates were created.

Our first aim was to describe the methodological approaches used to address racial misclassification of Indigenous people in population level, epidemiologic studies of mortality and health. Investigators have devised analytic methods to handle racial misclassification of Indigenous people, which usually manifests in 2 ways: they are misclassified with a non-Indigenous race (mostly as White or of European descent) or are missing race and ethnicity information altogether. In population-level studies where the goal is to estimate a mortality or morbidity rate, a prevalence, or risk, these misclassifications lead to undercounts of Indigenous people in numerators, and thus undercounted estimates due to deflated numerators.

For the remainder of this section, we provide results from US-based studies, though studies based outside current US borders are included in tables for reference, and interpretation of these results are woven throughout the discussion. We use the terms American Indian and Alaska Native to align with the terminology used in included research and data sets.

We identified 4 primary strategies investigators use to address misclassification: data linkage, geographic restriction, exclusion of Hispanic AI/AN, and producing several estimates (Table 5). A collection of other strategies is also used, which we categorized into a fifth, “other” category. Within the US borders, the most common approach to address misclassification is to use a data linkage (*n* = 76, 86%), whereby a gold standard data source thought to contain a definitive list of AI/AN is linked to another database containing the mortality or health data, like a cancer registry. This linkage allows for the identification of AI/AN who have been misclassified in the cancer registry as of a non-AI/AN race and then re-identifies them as AI/AN. The result of a linkage is usually an increase in an estimate, because additional AI/AN people have been identified and added to the numerator. The second analytic approach to address misclassification in numerator data is to restrict analyses to geographies where AI/AN misclassification is less common^1^ (*n* = 45, 51%). These areas include Contract Health Service Delivery Areas, now called Purchased Referred Care Designated Areas (PRCDAs), which, according to the US Census, have a larger proportion of AI/AN residing in them, compared with non-PRCDAs. Mortality and morbidity estimates are generally higher among AI/AN living in PRCDAs, compared with those living outside PRCDAs.

**Table 5:** Analytic methods used to address misclassification of indigenous people, by study location.

**Method to address misclassification**	**All studies** **(*n* = 97)**		**Non–US-based studies (*n* = 9)**	
	**No.**	**%** ^**a**^	**No.**	**%**
Data linkage	84	87	8	89
Geographic restriction	45	46	0	0
Exclude Hispanic AI/AN	26	27	0	0
Create several estimates	6	6	1	11
Other^b^	7	7	1	11
Use more than 1 method	48	49	1	11

Abbreviations: AI/AN, American Indian/Alaska Native; US, United States.

^a^ Percentages total more than 100% because studies could implement more than 1 method to address misclassification.

^b^ Other methods include imputation (*n* = 2 studies), electronic health record abstraction for language use (*n* = 1 study), source denominator and numerator data from the same data (*n* = 2 studies), and aggregate AI/AN and White individuals (*n* = 1 study).

Another set of analytic solutions to address Indigenous misclassification relates to denominators. For example, when estimating mortality rates, population counts from US Census data are used as rate denominators. Because of the single-race-bridging algorithms developed to harmonize race and ethnicity information over several years of Census data, Hispanic AI/AN are over-represented in single-race, bridged population counts.^1^^,^^29^ The result of this overinflated rate denominator is underestimated mortality and morbidity estimates. Restricting analyses to non-Hispanic AI/AN is 1 approach investigators use to avoid misclassification (*n* = 26, 30%), which usually results in an increased mortality or morbidity estimate because people have been removed from the denominator.

To address that there are numerous definitions that could be used to describe and then identify AI/AN people, 6% (*n* = 5) of studies used multiple data sources or approaches to calculate more than 1 mortality or health estimate. Another 6 studies (7%) used an approach not discussed above to address misclassification. Other methods included imputing data, abstracting language used from electronic health records, sourcing denominator and numerator data from the same data, and aggregating data on AI/AN and White individuals to create a combined estimate. Last, 53% (*n* = 47) of studies used a combination of methods to address misclassification.

Our second aim was to identify the limitations and strengths of the methodological approaches used to address racial misclassification of Indigenous people. We have identified 4 limitations. First, investigators combine data sources that use inconsistent processes and/or sources of race and ethnicity information. Second, several of the approaches used conflate the concepts of race, ethnicity, and nationality. Third, investigators are applying insufficient algorithms to bridge, impute, or link race and ethnicity information. Fourth, investigators assume AI/AN people have limited mobility. We discuss each of these limitations and note that they are not mutually exclusive.

### Limitation 1: Combining inconsistently collected race and ethnicity information

Different methods with different meanings are used by the data sources that monitor the burden of mortality or health collect information on race and ethnicity (Table 6). For example, decedent race and ethnicity on death certificates tend to be assigned by funeral directors, morticians, or a physician who is present upon death, with or without consultation with next of kin,^30^^,^^31^ whereas race and ethnicity in trauma registries may be self-reported, administratively reported, or provider reported. Unfortunately, investigators do not always articulate how race or ethnicity data are gathered or what these data mean. The biomedical sciences have already been criticized for this practice, which leads to over-reliance on genetic explanations for racial differences.^16^^,^^32^^–^^34^

**Table 6:** American Indian and Alaska Native identity information source, by data type (specific to the US context).

**Data set type**	**Who provides AI/AN identity information**	**Common use**
Indian Health Service hospital or facility registration or billing^a^	Any combination of hospital or facility administrative staff (e.g., admissions, check in) or self-report^b^	Disease numerator or linkage
Tribal health (includes 638 contract or compact) registration or billing^a^	Any combination of hospital or facility administrative staff (e.g., admissions, check in) or self-report^b^	Disease numerator or linkage
Urban Indian Health facility registration or billing	Self-report (if also a federally qualified health center)	Disease numerator or linkage
Hospital or facility (non–Indian Health Service) enrollment or billing	Any combination of hospital or facility administrative staff (e.g., admissions, check in) or self-report^b^	Disease numerator
Insurance claims	Often do not collect race data; might import from other data sources like the Social Security Administration or use imputation^b^	Disease numerator
Mortality data (death certificate)	Any combination of funeral directors, morticians, or physicians^b^	Mortality numerator
Disease or trauma registry	Any combination of diagnosing physicians or laboratories^b^	Disease numerator
Birth data (birth certificate)	Any combination of parents or birth and delivery nurses, physicians, staff^b^	Disease or birth rate numerator
Census counts	Self-report	Disease and mortality denominator

Abbreviation: AI/AN, American Indian/Alaska Native.

^a^ These data are not often considered or included in analyses; rather, individuals included in these data are assumed to be AI/AN.

^b^ Depends on local circumstances and context, practices and norms.

**Figure 2:**
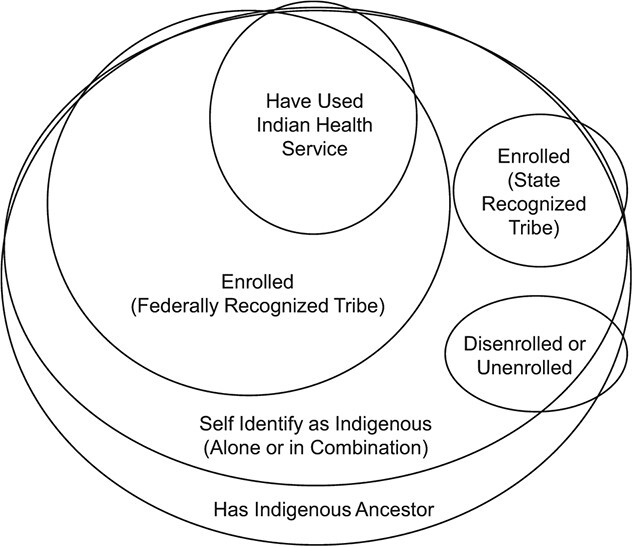
Areas of intersection for American Indian and Alaska Native identity in the United States. Areas of intersection, overlap, and nonoverlap in various conceptualizations of Indigenous identity in the United States context. Of note, circles and areas of overlap are not necessarily proportionally accurate.

Relatedly, investigators combine several data sources. The numerator relies on 1 source, typically death certificates or registry data, and the denominator on another, typically 1 of the population counts created by or in partnership with the US Census (e.g., American Community Survey; Surveillance, Epidemiology, and End Results). Often, 2 data sources collect race and ethnicity information through different methods, which makes it difficult to understand to whom the resulting estimates apply. For example, in estimating AI/AN mortality rates, the numerator might include individuals identified as AI/AN by a mortician and those data will then be linked with Indian Health Service (IHS) records to further identify some AI/AN who were misclassified as non-AI/AN on the death certificate. The denominator, on the other hand, will include individuals self-identifying as AI/AN. Although there is certainly overlap among people coded as AI/AN on a death certificate, who have received IHS services, and who self-identify as AI/AN, there is also variability in who will be identified as AI/AN through these data collection approaches (Figure 2). Furthermore, there has been little work to quantify the extent of nonoverlap among these 3 data collection sources. Investigators attempt to avoid the issue of mismatched numerator and denominator race and ethnicity information by using (1) numerator data that apply a similar single-race bridging algorithm as the Census-derived denominator data or (2) data sources in which race and ethnicity information is collected and reported the same way in the numerator and denominator.^35^^–^^37^

### Limitation 2: Conflating race, ethnicity, and nationality

The IHS system is responsible for providing health services to AI/AN enrolled in a federally recognized tribe.^38^ Direct care is provided in 37 states through federally managed IHS, tribal, and urban facilities, or through purchased referred care from non-IHS providers. The IHS serves more than 2.5 million of the 5.2 million AI/AN identified in the 2010 US Census.^39^ Anyone seen at a federally run IHS facility should be included in the IHS’s patient registration database. Among studies that use data linkages to correct for misclassification of AI/AN in the United States, 93% (*n* = 71 of 76) use IHS patient registration data.

However, IHS eligibility is not straightforward and, importantly, is not restricted to those racially identified or identifying as AI/AN (see Web Appendix for eligibility). Rather, exceptions are made for individuals who need emergency care, for preventing spread of communicable diseases, for non-AI/AN family members of AI/AN patients, for indigent people when the his can be reimbursed, and for non-AI/AN people pregnant with a fetus that may eventually identify as an AI/AN person.^40^ Descendants of those enrolled in a federally recognized tribe are also eligible. Every tribe has the right to set its citizenship criteria, which might be tied to, among other factors, blood quantum, residence, ties to community, and lineal descent; thus, the requirements for enrollment, and thus descendancy, vary by tribe. Regardless, studies that link IHS data to correct for misclassification rarely disaggregate by racial or ethnic identity those seen by the IHS. A notable exception is the study by Espey et al.,^41^ in which they clearly describe restricting IHS data to those identifying as AI/AN using an IHS-developed algorithm that considers blood quantum, beneficiary code, and tribe to confirm AI/AN identity.

In addition to providing care to non-AI/AN people, the IHS only provides care to a subset of AI/AN people. American Indian/Alaska Native people who are members of state-recognized tribes, live primarily in urban settings, live outside of PRCAs, or are otherwise not eligible for IHS will be underrepresented in IHS databases. Therefore, linkage with IHS data provides an imperfect solution because its use continues to undercount AI/AN people. Linkage to IHS data also subtly suggests that citizenship in a federally recognized tribe is the important metric by which to identify or define AI/AN people. Moreover, although AI/AN people have been racialized as part of the settler-colonial project, some AI/AN people also identify as citizens of Native nations, and citizenship criteria may or may not overlap with the AI/AN racialized identity. Use of IHS records for linkage, therefore, merges concepts of race, ethnicity, and nationality.

### Limitation 3: Applying insufficient algorithms to bridge, link, or impute race and ethnicity

Nearly every study estimating a health or mortality rate uses US Census–based, bridged, single-race population counts as denominators. In 1997, the OMB issued a revision to the 1977 race data collection standards to increase race reporting options from 4 to 5 categories and to allow individuals to select more than 1 racial identity. The OMB required all federal agencies to comply with the 1997 directive by 2003, meaning that during the transition, 2 incomparable race standards were used. In response, bridging methods were created to assign a single race category to individuals who had selected multiple racial identities. For example, the National Center for Health Statistics developed a method to bridge the 1997 OMB 5-race directive to the 1977 4-race directive. This algorithm was applied to the 2000 Census to make these data compatible with previous Censuses.^42^^,^^43^ The other issue addressed by bridging methods is to make numerator and denominator race-specific counts compatible.

Several bridging methods have been proposed.^42^^–^^44^ Regardless of the exact method, we identify some underlying, potentially meaningful implications of their use. First, bridging methods are applied only to multiracial individuals and, if the algorithms are problematic, the impact will be amplified for AI/AN because a greater proportion of AI/AN, compared with other racial groups, identify as multiracial. Second, bridging methods also make assumptions about how people might racially identify to assign a single race to multiracial individuals. For example, they assume that identity is not context dependent. However, we know that for AI/AN, identity can be fluid.^15^^,^^45^ Third, population-level data, such as from the National Health Interview Survey, are used to develop bridging models. These data may not adequately include a representative sample of AI/AN people and may only include a few hundred to 1000 AI/AN identifying individuals a year.^46^ This means commonly used bridging methods have been developed using a select subset of people that identify as AI/AN and may not represent the diverse experiences across Indian Country. Investigators ask people to identify their unique and multifaceted racial and ethnic identities, yet ignore these complexities in analyses, which further erodes the already precarious sense of trust between investigators and AI/AN communities.^47^

Studies using data linkage often use probabilistic linkage to identify observations in 2 data sources that represent the same person. Matching factors might include social security number, date of birth, surname, first name, or gender. The algorithms identify the potential match by assigning a score. The higher the score, the greater the probability that the match is a true match.^48^ However, the matching factors may be differentially missing for AI/AN people or for specific subsets of AI/AN individuals. For example, those married multiple times might be included with different last names in different data sets and, therefore, the data do not positively link.

Although probabilistic linkage software has made the process for finding potential matches easier, adjudication is necessary when matches are unclear. One protocol, for example, suggests that anyone with AI/AN in the first race field remains AI/AN for analysis. When the first race listed is White or is not listed (missing data), but there is a positive IHS link, the individual is reclassified as AI/AN. However, if the first race field is Black or Asian and there is a positive IHS link, the first race is retained.^49^ This means that individuals identifying as AI/AN and White will be categorized as AI/AN, whereas individuals identifying as AI/AN and Black will remain categorized as Black, reducing the number of people who are ultimately categorized as AI/AN.^16^^,^^50^ Although probabilistic linkage provides a replicable method to address misclassification, the exact algorithm and the adjudication process allow for biases to continue to play a role in reclassification.

Last, 2 studies used imputation to estimate an individual’s missing race, which is a problem in administrative health data.^51^ Imputation methods have been developed to improve Hispanic and Asian identification, particularly through use of surname and geographic area of residence.^52^^–^^54^ Validation studies suggest this imputation works well for those groups, yet these methods are applied to data containing information on AI/AN with limited success^55^^,^^56^ because common AI/AN surnames are not prioritized or included, AI/AN surnames are geographic or tribe specific, and AI/AN are a mobile population.^57^ Furthermore, imputation methods based on surname and geocoding may not be practical for multiracial individuals, which, again, include a greater proportion of AI/AN people.

### Limitation 4: Assuming hyperlocation of indigenous people

As a result of federal policy, like the Indian Relocation Act of 1953, AI/AN today largely live outside of reservation lands.^57^ Moreover, AI/AN are a mobile population that may travel for weeks or months for ceremonies or to fulfill family responsibilities. Data linkage with his or tribal rolls and imputation methods, specifically, do not recognize this mobility. Rather, the methods assume hyperlocality, or that Lummi people, for example, only live in Washington and AI/AN of other tribes do not live in these same locales. Data linkages wihisIHS are often done at the facility or state level, meaning Lummi living outside Washington or Lummi who do nohisse IHS are not included. Furthermore, data linkages with tribal rolls will often include just the citizens residing in the service area of the tribe. So again, a data linkage with the Lummi tribal roll will include just the Lummi living in Washington or a subset of counties within and exclude those living elsewhere. Relatedly, the traditional territory of the Lummi includes land that is currently part of Canada. These methods do not recognize that colonial geographic borders have been superimposed over borders defined by Indigenous people.

### Strengths

It is challenging to apportion resources to people who, in data, do not exist. Therefore, the strengths we mention here are strengths insofar as they result in increased resource allocation or creation of public policy to address relevant health concerns among AI/AN people. First, the new estimates of health and mortality are often viewed as more robust or reliable because they result in the identification of additional AI/AN people, which tends to increase rates and demonstrate greater disease and mortality burdens. Additionally, the methods used can result in an increase in disparity estimates or reveal new disparities.

Second, some studies used community-driven or community-engaged approaches to improve identification of Indigenous people, particularly in places whis poor IHS coverage or severely undercounted AI/AN populations, such as in urban areas, and in the southeastern United States. For example, Creswell et al.^58^ increased the number of AI/AN cancer cases counted in Wisconsin by 21.3% through a community-engaged approach with Tribal, urban, and public health stakeholders. Their study illustrates how, in addition to improving registry data and surveillance, epidemiologic studies can build capacity and contribute to Indigenous data-sovereignty goals. Collaborating with 7 state-recognized, non-federally recognized Tribes in North Carolina, Yankaskas et al.^59^ generated a linkage process between state cancer registries and Tribal membership rolls. These investigators found that nearly 18% of AI/AN were misclassified on the state cancer registries, and the linkage increased the AI/AN cancer rates by 41% for prostate, 18% for female breast, 10% for lung, and 11% for colorectal cancers. This approach, therefore, contributed to revealing AI/AN resource needs in an area not sehisd by the IHS. Foo et al.^60^ very specifically identified Mayan Indigenous people in 1 California county who otherwise would be invisible as an Indigenous population with particular needs. In collaboration with interpreters, investigators reviewed inpatient medical records for a Mayan language noted as first language, finding that “two-thirds of Maya patients were misclassified as native non-Indigenous Spanish speakers.”^58(p298)^ Because this population correlates to high-exposure employment contexts, this research helped target COVID-19 prevention, testing, and contact-tracing strategies. The community-engaged approach exemplified in these studies highlight how increased ownership of the research process produces richer, culturally competent, and inclusive results and, ultimately, more appropriately targeted resource investment.

## Discussion

We reviewed 97 articles from 3 databases to document methods used to address misclassification of Indigenous people in population-level health and mortality studies. Although several commentaries have been published on this topic, none included quantification of these methods or used a systematic process for identification of articles. Furthermore, the epidemiologic literature abounds with discussion of methods, whereas work on methodologies, particularly in Indigenous contexts, remain overlooked. We draw a distinction between methods and methodologies such that methods includes the tools of analyses (e.g., multiple imputation) and methodologies include the way we do our work (e.g., how we collect data, what interpretations we elicit).^61^^,^^62^ This scoping review should prompt conversation about epidemiologic methods and methodologies.

Several methods are used to address Indigenous misclassification, the most common of which are hisnkages with IHS data. Despite being a popular method that has changed the landscape of knowledge of the burdens of disease and mortality for AI/AN communities, these linkages perpetuate the exclusion of AI/AN people. Moreover, this method contributes to a conflation of race and nationality while also furthering a narrative of Indigenous belonging that prioritizes colonial logics of who is identified as AI/AN.

Included studies predominantly focused on estimating cancer or mortality burdens, which may reflect the data sources readily available for population-level epidemiologic studies rather than a heightened interest in these outcomes among AI/AN communities. Also, although comparisons to dominant populations highlight significant disparities, the centering of data for White populations (70% of studies) may perpetuate narratives of deficiency and decontextualized understandings of Indigenous health.^63^ Moreover, the White comparative group is rarely critically deconstructed and is unproblematically assumed as a homogeneous, stable, comparison group.

The complexities of colonial-state power in defining who is Indigenous and how this has shifted over time may influence data on Indigenous status. This has not been sufficiently addressed in the epidemiologic literature for the United States, as it has been examined in Canada or Australia. There, Indigenous scholars have explained the need to consider the historical trajectories and political goals of nation-states in shaping who does and does not count as Indigenous, and to innovate methodologies in ways that do not harm Indigenous peoples. Elias et al.,^64^ for example, situate weaknesses in Indigenous-group identifiers in Canadian administrative databases in the context of the historical and ongoing colonial construction of “the Indian.” This category has been largely shaped by settler-state objectives of assimilation and elimination of Indigenous people. Their legal review reveals how much colonial-state approaches to identification of Indigenous people are at odds with Indigenous ones that center familial kinship. Nonetheless, even as an “administrative artifact created to track Indian status [the federal registry] has the greatest data linkage potential” when taking an “inclusive familial kinship approach…to shift data interpretation to reflect Indigenous heritage…[and generate] innovative data linkage programs…critical to ensure that all indigenous peoples are properly counted, avoiding the nihilistic outcome of administrative attrition.”^62(p185)^

Similarly in Australia, Griffiths et al.^65^ detailed the transformation of definitions of Indigenous people through 3 eras across the 20th century from the blood quantum era to the race era, to the current 3-part definition era, which centers on origin. Although additional robust health and well-being data collection mechanisms were instituted in the 1990s, Griffiths et al.^63^ identify issues with varying quality and incompleteness. They further contextualize these issues in ongoing colonial tensions and the need for Indigenous data governance. Williamson et al.^66^ describe a history of purposeful colonial shifts in the definition of Indigenous people and their exclusion from participation in the US Census, from being perceived as a “dying race”^66^ with no future in the nation-state, to Indigenous people themselves perceiving no relevance or meaning in participating.

Over time, this colonial context has constructed the Indigenous category in data sets as 1 of racial origin rather than of identification with present Indigenous communities and languages. Without the engagement of Indigenous people in the process to improve how data are collected, data sets are biased in ways that can lead to significant rifts between people differentially situated in the colonial hierarchies of Indigeneity. Biddle and Markham^67^ found that the well-intentioned open option for self-identification on the 2016 Australian Census increased the number of people identified as Indigenous by nearly 14% in the 5-year period since the previous Census. Some noted that this increase could not be accounted for by natural population growth, and that the bulk of new identifiers were in urban areas and had higher living standards, prompting debates about the authenticity of “box checkers” and the implications this may have in generating a false picture of improvement in the overall health status of Indigenous people. A qualitative study by Watt and Kowal^68^ offers a nuanced analysis of new identifiers for whom taking up Indigenous identification brings “a sense of deep belonging to the Australia continent, a holistic spiritualism, and a meaningful family history,”^68^^(p77)^ which the authors highlight as significant for understanding how changing practices of identification factor in population change.

Williamson et al.^66^ and Crooks et al.^69^ assert a fundamental difference between origin and identity. Williamson et al. write, “being of origin in-and-of-itself, whilst necessary, is insufficient.”^64(p6)^ They suggest adding questions to the Census that clarify specific identification with Indigenous nations and languages, as is done in New Zealand and, to a degree, on the US Census, which leaves a blank line to “print the name of the enrolled or principal tribe.” In the United States, the OMB’s definition of AI/AN includes reference to tribal affiliation or community attachment, without defining what this means. Is affiliation the same as enrollment? What is community attachment and how much is enough to claim it? In the US context, people report their AI/AN background differently, depending on whether they are asked about their ancestry or their race.^70^^–^^72^ Moreover, AI/AN people with parents of different racial identities—1 AI/AN and 1 not—are more likely to report a single race rather than multiple racial identities, which is different than the trends among people without an AI/AN parent.^45^ So, although self-reporting of race is often considered the gold standard practice for data collection, not all measures of self-reported AI/AN status are the same, and some might be more appropriate than others, given the research context.

Scholars argue for a paradigm shift not only for how Indigenous data are defined but also how they are collected, owned, and used under Indigenous governance, to minimize harm, and to be applied to Indigenous community planning for health and well-being. As detailed earlier, this paradigm shift requires deep decolonization and Indigenization of quantitative methodologies through community partnerships and careful localized data validation steps,^72^ as developed in the Tribal Epidemiology Centers^63^ and described by Hayward et al.^74^ for several recent projects in Canada. These efforts combine with community-based participatory methods to accomplish several objectives, including building capacity, shifting standards to align with Indigenous experiences, developing culturally appropriate data collection practices, and “distinct epidemiological methods based on Indigenous knowledge systems,”^74^ which center local and regional contexts. These yield different types of results than ones oriented toward a scalable colonial national standard, but more accurately capture diversity within a region to provide Indigenous nations and communities with useable information in self-determined health care provision and prevention, and in their relationships with state agencies.

The application of colonial logics of identity and belonging in the data sets uncritically drawn on by epidemiologists diminishes both the authority and legitimacy of Indigenous epistemes, and ascribes racialized and hierarchized power within colonial systems and institutions, which, in turn, govern citizenship and the allocation of resources. Investigators are thus exercising not only a form of unconscious bias but they are also contributing with powerful effect to determinations of who does and does not belong as Indigenous people.

### Limitations

The processes used to identify articles have limitations. Our search terms, including use of “misclassification” and “underestimation,” for example, may limit the utility of the searches by restricting results to studies identifying problematic misclassification. There are projects however, that address misclassification but do not frame their work that way. The Canadian “Our Health Counts” project^75^ used respondent-driven sampling to demonstrate considerable undercount of Indigenous people in urban areas by the Canadian Census while also documenting persistent health disparities for Indigenous people.^76^^,^^77^ Our search strategy did not identify articles from this project despite the relevance of its analytic approach. As a result, this scoping review may unintentionally exclude studies that address misclassification through community-based methods or that use localized, culturally appropriate approaches to data collection. Relatedly, we used the term “Indigenous” and included key-word synonyms like “American Indian,” “Alaska Native,” “Aboriginal,” “Native Hawaiian,” or “Pacific Islander.” If a study was localized to a specific tribal community and used the tribe’s name as a keyword, rather than these less descriptive terms, the article may not have been identified through our search. Furthermore, the indexing practices of the National Library of Medicine might be insufficient for the purposes of this scoping review. We found Medical Subject Heading terms associated with methods, statistics, and Indigenous identities to be potentially underused. We added 22 articles after reviewing included article reference lists, which potentially indicates that the search terms may be insufficient, the indexing of databases insufficient, or both.

Notably, there were 2 journals, *Cancer* and the *American Journal of Public Health*, that each published a special issue on methods to address misclassification of AI/AN people. Most of the articles included data linkages with IHS data, and these special issues, in part, account for the high frequency of IHS data linkages described by our results. Furthermore, nationwide efforts have been made to link death certificate data and cancer registry to IHS records, which are then harvested to produce several papers on the topic of AI/AN mortality or cancer burden. To determine if these 2 issues biased our results, we recalculated the distribution of methods used to address misclassification presented in Table 5, whereby we included just 1 article using the Surveillance, Epidemiology, and End Results/National Program of Cancer Registries/IHS data in the final count and excluded all the others. The same was done for the National Vital Statistics System/IHS linkage and other similar linkages. Despite this, data linkages remain the most used method (67%) to address misclassification of AI/AN people, with 76% of those linking to IHS data.

### Conclusion

Disciplinary self-reflection acknowledges the ways in which disciplinary norms work to replicate inequalities or enable the settler-colonial project,^78^ and builds toward a public health critical race praxis.^79^ There is no perfect solution to the issue of Indigenous misclassification in population-based studies; however, we suggest investigators take heed of data sovereignty conversations^80^ and Indigenous people offering suggestions for improvement^8^^,^^63^^,^^81^ so Indigenous people do not continue to disappear in data. Moreover, investigators should make informed decisions based on the strengths and weaknesses of possible misclassification approaches and clearly articulate their decision-making processes. The United Nations and scholars^82^ recommend using methods that help identify Indigenous people rather than define them. Identity is multifaceted, but when examined, measured, and analyzed with community taking the lead, it can yield transformative results.^83^

## Acknowledgements

We thank the audiences at the 2022 annual meetings of the Interdisciplinary Association for Population Health Sciences and the Society for Epidemiologic Research and the participants of Michigan State University’s Center for Excellence in Diversity in Medicine for their feedback on early versions of this article.

This work was presented in part at the Society for Epidemiologic Research annual meeting, Chicago, Illinois, June 14–17, 2022; and the Interdisciplinary Association for Population Health Science annual meeting, Minneapolis, Minnesota, September 20–23, 2022.

## Supplementary material

Supplementary material is available at *Epidemiologic Reviews* online.

## Funding

Danielle Gartner’s time was supported through funds provided by Michigan State University’s Center for Excellence in Diversity in Medicine.

## Conflict of interest

Authors have no conflicts of interest to declare.

## Disclaimer

The views expressed in this article are those of the authors.

## Data availability

The data are available from the corresponding author.

## Supplementary Material

Web_Material_mxad001
