# Supplementary material for: Misracialization of Indigenous people in population health and mortality studies: a scoping review to establish promising practices
Source: Epidemiol Rev. 2023 Apr 6;45(1):63–81. doi: 10.1093/epirev/mxad001 (PMC10748801; doi:10.1093/epirev/mxad001)
Supplement: Web_Material_mxad001 [file web_material_mxad001.docx]

**Web Material**

Misracialization of Indigenous people in population health and mortality studies: A scoping review to establish promising practices

Danielle R. Gartner, Ceco Maples, Madeline Nash, Heather Howard-Bobiwash

**Table of Contents:**

Web Appendix

**Web Appendix: Indian Health Service Eligibility**

IHS services are free for eligible American Indians and Alaska Natives. However, not all AI/AN are eligible. Instead, IHS services are limited to persons of “American Indian and/or Alaska Native (AI/AN) descent and belongs to the Indian community served by the IHS program” ^1(secs2-1.2)^. This status is evidenced by: (i) “Membership, enrolled or otherwise, in an AI/AN Federally-recognized Tribe or Group”, (ii) residence on tax-exempt land or ownership of restricted property, (iii) active participation in tribal affairs”, or (iv) “Any other reasonable factor indicative of Indian decent” ^1(secs2-2.1)^. Exceptions are made for individuals in need of emergency medical care. IHS is also available for non-AI/AN family members and for non-Indian women pregnant with an AI/AN child ^2^.

Furthermore, the Patient Protection and Affordable Care Act (ACA), which updated and permanently reauthorized the Indian Health Care Improvement Act, adjusted IHS eligibility criteria. A Congressional Research Service report^3^ includes the following summary of eligibility changes following the passage of the ACA:

“Section 194. Health Services for Ineligible Persons: This section amends IHCIA Section 813 [25 U.S.C. §1680c] with new language that authorizes IHS health services for certain otherwise ineligible persons, including spouses or children of eligible Indians, non-Indian women carrying Indian babies, or persons in need of emergency stabilization, or for prevention of communicable diseases. The section authorizes the governing body of Indian tribes operating health facilities under ISDEAA contracts to determine whether to provide services to ineligible persons. The section also sets criteria for providing services, such as requiring reimbursement and tribal approval, and directs that reimbursements, including under Medicare or Medicaid, be credited to the facility providing the service and be available for expenditure by the facility. The section permits the Secretary to provide services to indigent individuals who are not otherwise eligible for IHS services provided that the state or local government agrees to reimburse IHS for providing this service. The section also permits extending hospital privileges to non-IHS health care practitioners who provide service to certain ineligible persons.” ^3(p32)^

**References**

1. Chapter 1 - Eligibility for Services | Part 2. The Indian Health Manual (IHM). Published June 28, 2017. Accessed March 30, 2023. https://www.ihs.gov/ihm/pc/part-2/chapter-1-eligibility-for-services/

2. Indian Health Service. The Indian Health Manual (IHM). Published online June 9, 2020. https://www.ihs.gov/ihm/

3. Heisler EJ. *The Indian Health Care Improvement Act Reauthorization and Extension as Enacted by the ACA: Detailed Summary and Timeline*. Washington, DC: Congressional Research Service; 2014.
